# Counter-Regulatory Response to a Fall in Circulating Fatty Acid Levels in Rainbow Trout. Possible Involvement of the Hypothalamus-Pituitary-Interrenal Axis

**DOI:** 10.1371/journal.pone.0113291

**Published:** 2014-11-18

**Authors:** Marta Librán-Pérez, Cristina Velasco, Marcos A. López-Patiño, Jesús M. Míguez, José L. Soengas

**Affiliations:** Laboratorio de Fisioloxía Animal, Departamento de Bioloxía Funcional e Ciencias da Saúde, Facultade de Bioloxía, Universidade de Vigo, Vigo, Spain; Institut National de la Recherche Agronomique (INRA), France

## Abstract

We hypothesize that a decrease in circulating levels of fatty acid (FA) in rainbow trout *Oncorhynchus mykiss* would result in the inhibition of putative hypothalamic FA sensing systems with concomitant changes in the expression of orexigenic and anorexigenic factors ultimately leading to a stimulation of food intake. To assess this hypothesis, we lowered circulating FA levels treating fish with SDZ WAG 994 (SDZ), a selective A1 adenosine receptor agonist that inhibits lipolysis. In additional groups, we also evaluated if the presence of intralipid was able to counteract changes induced by SDZ treatment, and the possible involvement of the hypothalamus-pituitary-interrenal (HPI) axis by treating fish with SDZ in the presence of metyrapone, which decreases cortisol synthesis in fish. The decrease in circulating levels of FA in rainbow trout induced a clear increase in food intake that was associated with the decrease of the anorexigenic potential in hypothalamus (decreased POMC-A1 and CART mRNA abundance), and with changes in several parameters related to putative FA-sensing mechanisms in hypothalamus. Intralipid treatment counteracted these changes. SDZ treatment also induced increased cortisol levels and the activation of different components of the HPI axis whereas these changes disappeared in the presence of intralipid or metyrapone. These results suggest that the HPI axis is involved in a counter-regulatory response in rainbow trout to restore FA levels in plasma.

## Introduction

Specialized neurons within mammalian hypothalamus have been suggested to detect increases in plasma levels of long-chain fatty acid (LCFA), but not short-chain (SCFA) or medium-chain (MCFA) FA through several mechanisms [1], [2], [3], such as i) FA metabolism through inhibition of carnitine palmitoyltransferase 1 (CPT-1) to import FA-CoA into the mitochondria for oxidation; ii) binding to FA translocase (FAT/CD36), and further modulation of transcription factors like peroxisome proliferator-activated receptor type α (PPARα), and sterol regulatory element-binding protein type 1c (SREBP1c); iii) activation of protein kinase C-θ; and iv) mitochondrial production of reactive oxygen species (ROS) by electron leakage resulting in an inhibition of ATP-dependent inward rectifier potassium channel (K_ATP_) activity. Changes in these systems have been associated [4] with the modulation of hypothalamic homeobox domain transcription factor (BSX), forkhead box 01 (Fox01), and phosphorylated cAMP response-element binding protein (pCREB). The action of these factors would result in the inhibition of the orexigenic factors agouti-related protein (AgRP) and neuropeptide Y (NPY), and the enhancement of the anorexigenic factors pro-opio melanocortin (POMC) and cocaine and amphetamine-related transcript (CART) ultimately leading to decreased food intake [1], [4].

In fish, a reduced food intake has been observed after feeding fish with lipid-enriched diets or in fish containing high fat stores [5], [6], [7], [8], [9], [10], [11] raising the question whether lipid sensing mechanisms regulating food intake may be also present in fish [12], [13]. Accordingly, we observed in rainbow trout *Oncorhynchus mykiss* that intraperitoneal [14] or intracerebroventricular [15] administration of oleate (LCFA) or octanoate (MCFA) elicited an inhibition in food intake. Furthermore, the treatment induced a response in the hypothalamus compatible with FA sensing including reduced potential of lipogenesis and FA oxidation, decreased potential of K_ATP_, and modulation of FAT/CD36 with subsequent changes in the expression of transcription factors [14], [15], [16]. This response is comparable in general with that reported in mammals with the main difference of the capacity of fish to respond to increased levels of an MCFA like octanoate [13]. Changes in these hypothalamic pathways can be also related to the control of food intake, since changes in mRNA levels of neuropeptides such as NPY and POMC-A1 were also noted [14], [15], [16]. In the hypothalamus of another fish species, the orange-spotted grouper (*Epinephelus coioides*), the involvement of FA metabolism and mitochondrial activity in the orexigenic effects of NPY has been also suggested [17].

The fall of blood glucose levels is sensed in central glucosensor areas eliciting counter-regulatory responses to restore glucose levels as demonstrated in mammals [18] and fish [19], [20]. This mechanism has been evidenced in rat for metabolites other than glucose, such as FA. Thus, the counter-regulatory response to decreased circulating levels of FA has been associated with the activation of the hypothalamus-pituitary-adrenal (HPA) axis, and, therefore, to enhanced circulating levels of glucocorticoids whose lipolytic action would restore plasma FA levels [21], [22], [23]. In fish fed with diets containing low lipid levels, an increase in food intake has been described [6], [8], [9], [10], [24], [25] but to date there is no evidence available regarding the existence of counter-regulatory responses to decreased FA levels. We hypothesize that the decrease in circulating levels of FA in rainbow trout would result in the down-regulation of putative hypothalamic FA sensing systems [13] with concomitant changes in the expression of orexigenic and anorexigenic factors ultimately leading to a stimulation of food intake. To assess this hypothesis, we lowered circulating FA levels in rainbow trout treating fish with SDZ WAG 994 (SDZ), a selective A1 adenosine receptor agonist that inhibits lipolysis [26]. We also evaluated if the presence of intralipid (a lipid emulsion of phospholipid-stabilized soybean oil for intravenous administration, Sigma Chemical Co) was able to counteract changes induced by SDZ treatment. Furthermore, we also evaluated the possible involvement of the hypothalamus-pituitary-interrenal (HPI) axis (fish equivalent to mammalian HPA) in the counter-regulatory response. Thus, we modified an accessible part of the HPI axis such as cortisol levels by treating fish with SDZ in the presence of metyrapone, which decreases cortisol synthesis in fish [27], [28].

## Materials and Methods

### Ethics statement

The experiments described comply with the Guidelines of the European Union Council (2010/63/UE), and of the Spanish Government (RD 55/2013) for the use of animals in research. The Ethics Committee of the Universidade de Vigo approved the procedures.

### Fish

Rainbow trout obtained from a local fish farm (A Estrada, Spain) were maintained for 1 month in 100 litre tanks under laboratory conditions and 12L:12D photoperiod in dechlorinated tap water at 15°C. Fish weight was 99±3 g. Fish were fed once daily (09.00 h) to satiety with commercial dry fish pellets (Dibaq-Diproteg SA, Spain).

### Experimental design

Following acclimation, fish were fasted for 24 h before treatment to ensure fish had basal levels of metabolic hormones including cortisol. On the day of experiment, a first set of fish were anaesthetized in tanks with 2-phenoxyethanol (Sigma, 0.2% v/v), and weighed. Then, 15 fish per group received intraperitoneally (IP) 10 mL.Kg^−1^ injection of saline solution alone (control, C), or containing SDZ (SDZ; Tocris, 60 µg.Kg^−1^), metyrapone (M; Sigma, 1 mg.Kg^−1^), both SDZ and metyrapone (SDZ+M), or both SDZ and intralipid (SDZ+IL; Sigma I-141, 3 mL.Kg^−1^). Blood, hypothalamus, and head kidney samples were taken 6 h after treatment, which was chosen on the basis of previous studies in which such time period was necessary to achieve changes in the FA sensing mechanisms when levels of FA were increased [14], [16]. Initial concentrations of SDZ were selected based on studies carried out previously in mammals [22], [29], and then in preliminary studies (data not shown) we evaluated different SDZ doses. Since SDZ is known to reduce mean arterial pressure and heart rate at high doses [29] we selected a dose (60 µg.Kg^−1^) able to lower levels of circulating FA without inducing any other apparent alteration. The concentrations of metyrapone and intralipid were selected based on previous studies carried out in rainbow trout [27], [28], [30] and mammals [22], respectively. In each group, 10 fish were used to assess enzyme activities and metabolite levels whereas the remaining 5 fish were used for the assessment of mRNA levels by qRT-PCR. In each sampling, fish were anesthesized as above, and blood was collected from the caudal vein with a heparinised syringe. Fish were then sacrificed by decapitation, and hypothalamus and head kidney (area containing interrenal cells, i.e. those involved in glucocorticoid synthesis in fish) were taken and stored as previously described [31], [32], [33].

In a second set of fish, we evaluated changes in food intake after IP administration of SDZ or SDZ+intralipid. Fish were randomly assigned to experimental groups in different tanks and fasted for 24 h before injection. Then, 8 fish per group were anesthesized and IP injected as above. Food intake was assessed 3 days before treatment (to define baseline data), and then 6 and 24 h after treatment. After feeding, the food uneaten remaining at the bottom (conical tanks) and feed waste were withdrawn, dried and weighed. The amount of food consumed by all fish in each tank was calculated as previously described as the difference from the feed offered [31], [32], [34]. Results are shown as the mean ± SEM of the data obtained in three different tanks per treatment.

### Assessment of metabolite levels and enzyme activities

Levels of FA, total lipid, triglyceride, glucose, and lactate in plasma were determined enzymatically using commercial kits (Wako for FA; Spinreact for total lipid, triglyceride, and lactate; Biomérieux for glucose) adapted to a microplate format. Plasma cortisol levels were assessed by ELISA using a commercially available kit (Cayman).

Samples used to assess hypothalamic metabolite levels were homogenized immediately by ultrasonic disruption in 7.5 vols of ice-cooled 0.6 M perchloric acid, and neutralized (using 1 M potassium bicarbonate). The homogenate was centrifuged (10,000 g), and the supernatant used to assay tissue metabolites. Tissue FA, total lipid, and triglyceride levels were determined enzymatically using commercial kits as described above for plasma samples.

Samples for enzyme activities were homogenized by ultrasonic disruption with 9 vols of ice-cold-buffer consisting of 50 mM Tris (pH 7.6), 5 mM EDTA, 2 mM 1,4-dithiothreitol, and a protease inhibitor cocktail (Sigma). The homogenate was centrifuged (10,000 g) and the supernatant used immediately for enzyme assays. Enzyme activities were determined using a microplate reader INFINITE 200 Pro (Tecan) and microplates. Reaction rates of enzymes were determined by the increase or decrease in absorbance of NAD(P)H at 340 nm or, in the case of CPT-1 activity, of 5,5′-Dithiobis(2-nitrobenzoic acid)-CoA (DTNB) complex at 412 nm. The reactions were started by the addition of supernatant (15 µl) at a pre-established protein concentration, omitting the substrate in control wells (final volume 265–295 µl), and allowing the reactions to proceed at 20°C for pre-established times (3–10 min). Enzyme activities are expressed per protein level, which was assayed according to the bicinchoninic acid method with bovine serum albumin (Sigma) as standard. Enzyme activities were assessed at maximum rates determined by preliminary tests to determine optimal substrate concentrations. ATP-citrate lyase (ACLY, *EC* 4.1.3.8) activity was assessed in a tris-HCl buffer (50 mM, pH 7.8) containing 100 mM KCl, 10 mM MgCl_2_, 20 mM citrate, 10 mM β-mercaptoethanol, 5 mM ATP, 0.3 mM NADH, 7 U.ml^−1^ malate dehydrogenase, and 50 µM Coenzyme A (omitted for controls). Fatty acid synthase (FAS, *EC* 2.3.1.85) activity was assessed in a phosphate buffer (100 mM, pH 7.6) containing 0.1 mM NADPH, 25 µM Acetyl-CoA, and 30 µM Malonyl-CoA (omitted for controls). Hydroxyacil-CoA dehydrogenase (HOAD, *EC* 1.1.1.35) activity was assessed in a imidazole buffer (50 mM, pH 7.6) containing 0.15 mM NADH and 3.5 mM Acetoacetyl-CoA (omitted for controls). CPT-1 (*EC* 2.3.1.21) activity was assessed in a tris-HCl buffer (75 mM, pH 8.0) containing 1.5 mM EDTA, 0.25 mM DTNB, 35 µM palmitoyl CoA, and 0.7 mM L-carnitine (omitted for controls).

### mRNA abundance analysis by quantitative RT-PCR

Total RNA extracted from tissues using Trizol reagent (Life Technologies) was treated with RQ1-DNAse (Promega). 4 µg total RNA were reverse transcribed into cDNA using Superscript II reverse transcriptase (Promega) and random hexaprimers (Promega). Gene expression levels were determined by real-time quantitative RT-PCR (q-PCR) using the iCycler iQ (BIO-RAD). Analyses were performed on 1 µl cDNA using the MAXIMA SYBRGreen qPCR Mastermix (Thermo Fisher Scientific), in a total PCR reaction volume of 25 µl, containing 50–500 nM of each primer. mRNA abundance of transcripts 3β-hydroxysteroid dehydrogenase (3βHSD), 11β-hydroxylase (11βH), acetyl-CoA carboxylase (ACC), ACLY, CART, corticotrophin releasing factor (CRF), corticotrophin releasing factor binding protein (CRFBP), FAT/CD36, CPT-1, citrate synthetase (CS), FAS, inward rectifier K^+^ channel pore type 6.x-like (Kir6.x-like), liver X receptor α (LXRα), malonyl CoA dehydrogenase (MCD), NPY, cytochrome P450 cholesterol side chain cleavage (P450scc), POMC-A1, PPARα, SREBP1c, steroidogenic acute regulatory protein (StAR), sulfonylurea receptor-like (SUR-like), and mitochondrial uncoupling protein 2a (UCP2) was determined as previously described in the same species [35], [36], [37], [38], [39], [40], [41], [42], [43], [44], [45]. Sequences of the forward and reverse primers used for each gene expression are shown in Table 1. Relative quantification of the target gene transcripts was done using elongation factor 1α (EF-1α) gene expression as reference, which was stably expressed in this experiment.

**Table 1:** Nucleotide sequences of the PCR primers used to evaluate mRNA abundance by RT-PCR (qPCR).

	Forward primer	Reverse primer	Data base	Accession Number
3β-HSD	TCACAGGGTCAACGTCAAAG	CCTCCTTCTTGGTCTTGCTG	GenBank	S72665.1
11βH	ATTTGCCCTGTACGAGTTGG	GGATGATGATGTCTCTGACTG	GenBank	AF179894
ACC	TGAGGGCGTTTTCACTATCC	CTCGATCTCCCTCTCCACT	Sigenae	tcbk0010c.b.21_5.1.om.4
ACLY	CTGAAGCCCAGACAAGGAAG	CAGATTGGAGGCCAAGATGT	GenBank	CA349411.1
CART	ACCATGGAGAGCTCCAG	GCGCACTGCTCTCCAA	GenBank	NM_001124627
CPT-1c	CGCTTCAAGAATGGGGTGAT	CAACCACCTGCTGTTTCTCA	GenBank	AJ619768
CPT-1d	CCGTTCCTAACAGAGGTGCT	ACACTCCGTAGCCATCGTCT	GenBank	AJ620356
CRF	ACAACGACTCAACTGAAGATCTCG	AGGAAATTGAGCTTCATGTCAGG	GenBank	AF296672
CRFBP	GGAGGAGACTTCATCAAGGTGTT	CTTCTCTCCCTTCATCACCCAG	GenBank	AY363677
CS	GGCCAAGTACTGGGAGTTCA	CTCATGGTCACTGTGGATGG	Tigr	TC89195
EF-1α	TCCTCTTGGTCGTTTCGCTG	ACCCGAGGGACATCCTGTG	GenBank	AF498320
FAS	GAGACCTAGTGGAGGCTGTC	TCTTGTTGATGGTGAGCTGT	Sigenae	tcab0001c.e.06 5.1.s.om.8
FAT/CD36	CAAGTCAGCGACAAACCAGA	ACTTCTGAGCCTCCACAGGA	DFCI	AY606034.1
Kir6.x-like	TTGGCTCCTCTTCGCCATGT	AAAGCCGATGGTCACCTGGA	Sigenae	CA346261.1.s.om.8:1:773:1
LXRα	TGCAGCAGCCGTATGTGGA	GCGGCGGGAGCTTCTTGTC	GenBank	FJ470291
MCD	TCAGCCAGTACGAAGCTGTG	CTCACATCCTCCTCCGAGTC	Sigenae	BX869708.s.om.10
NPY	CTCGTCTGGACCTTTATATGC	GTTCATCATATCTGGACTGTG	GenBank	NM_001124266
P450scc	ATGCGTCAGGACACTAACAC	CAGCGGTATCATCTTCAGCA	GenBank	S57305.1
POMC-A1	CTCGCTGTCAAGACCTCAACTCT	GAGTTGGGTTGGAGATGGACCTC	Tigr	TC86162
PPARα	CTGGAGCTGGATGACAGTGA	GGCAAGTTTTTGCAGCAGAT	GenBank	AY494835
SREBP1c	GACAAGGTGGTCCAGTTGCT	CACACGTTAGTCCGCATCAC	GenBank	CA048941.1
StAR	CTCCTACAGACATATGAGGAAC	GCCTCCTCTCCCTGCTTCAC	GenBank	AB047032
SUR-like	CGAGGACTGGCCCCAGCA	GACTTTCCACTTCCTGTGCGTCC	Sigenae	tcce0019d.e.20_3.1.s.om.8
UCP2a	TCCGGCTACAGATCCAGG	CTCTCCACAGACCACGCA	GenBank	DQ295324

3βHSD, 3β-hydroxysteroid dehydrogenase; 11βH, 11β-hydroxylase; ACC, Acetyl-CoA carboxylase; ACLY, ATP-citrate lyase; CART, cocaine- and amphetamine-related transcript; CPT-1, carnitine palmitoyl transferase type 1; CRF, corticotrophin releasing factor; CRFBP, corticotrophin releasing factor binding protein; CS, citrate synthetase; EF-1α, elongation factor 1α; FAS, fatty acid synthetase; FAT/CD36, fatty acid translocase; Kir6.x-like, inward rectifier K^+^ channel pore type 6.x-like; LXRα, liver X receptor α; MCD, malonyl CoA dehydrogenase; NPY, neuropeptide Y; P450scc, cytochrome P450 cholesterol side chain cleavage; POMC-A1, pro-opio melanocortin A1; PPARα, peroxisome proliferator-activated receptor type α; SREBP1c, sterol regulatory element-binding protein type 1c; StAR, steroidogenic acute regulatory protein; SUR-like, sulfonylurea receptor-like; UCP2a, mitochondrial uncoupling protein 2a.

Thermal cycling was initiated with incubation at 95°C for 15 min using hot-start iTaq DNA polymerase activation; 40 steps of PCR were performed, each one consisting of heating at 95°C for 15 s for denaturing, annealing at specific temperatures for 30 s, and extension at 72°C for 30 s. Following the final PCR cycle, melting curves were systematically monitored (55°C temperature gradient from 55 to 95°C) to ensure amplification of only one fragment. Each sample was assessed in triplicate. Samples without reverse transcriptase and samples without RNA were run for each reaction as negative controls. Only efficiency values between 85–100% were accepted (the R^2^ for all the genes assessed was always higher than 0.985). Relative quantification of the target gene transcript with the EF-1α reference gene transcript was made following the Pfaffl method [46].

### Statistics

Comparisons among groups were carried out with two-way ANOVA with treatment and time as main factors for food intake data whereas the remaining parameters were compared with one-way ANOVA. Post-hoc comparisons were carried out with a Student-Newman-Keuls test, and differences were considered statistically significant at *P*<0.05.

## Results

**Figure 1:**
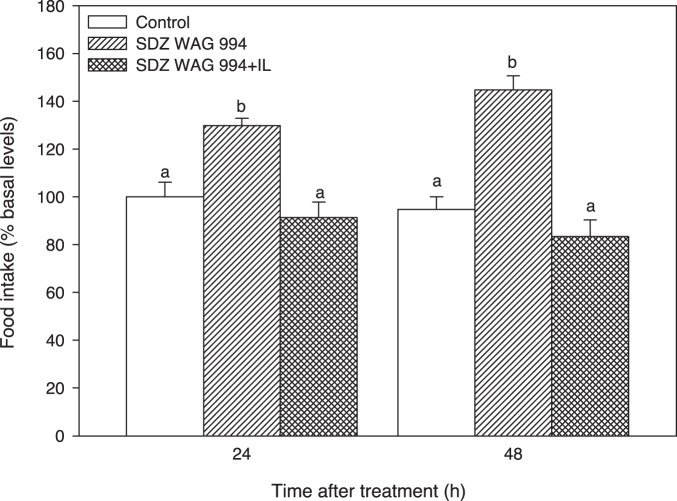
Changes in food intake after intraperitoneal treatment. Food intake of rainbow trout after 6 h or 24 h of intraperitoneal administration of 10 mL.Kg^−1^ of saline solution alone (control) or containing SDZ WAG 994 alone (60 µg.Kg^−1^) or containing SDZ WAG 994 (60 µg.Kg^−1^) together with intralipid (3 mL.Kg^−1^) solution. Different letters indicate significant differences (*P*<0.05) among treatments at the same time. Food intake is shown as mean+S.E.M. of the percentage of food ingested with respect to basal levels (calculated as the average of food intake the three days previous to experiment). The results are shown as mean+S.E.M. of the results obtained in three different tanks in which 8 fish were used per group in each tank. Different letters indicate significant differences (*P*<0.05) among treatments at each time.

Food intake increased after 6 and 24 h of treatment with SDZ, and the presence of intralipid counteracted the increase (Fig. 1).

**Figure 2:**
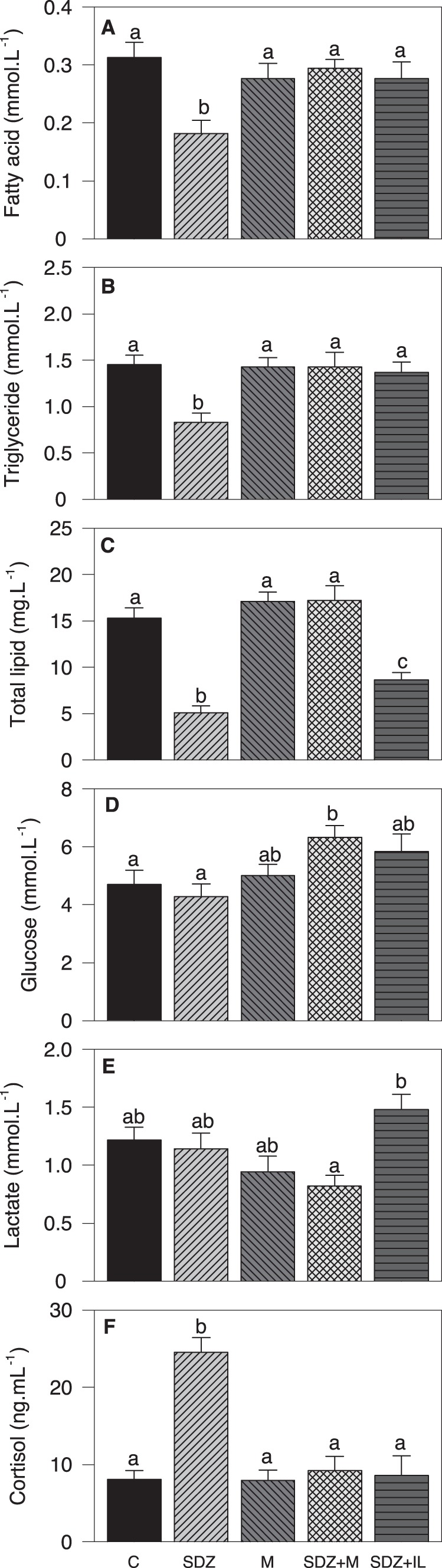
Changes in the levels of plasma metabolites after intraperitoneal treatment. Levels of fatty acid (A), triglyceride (B), total lipid (C), glucose (D), lactate (E), and cortisol (F) in plasma of rainbow trout after 6 h of intraperitoneal administration of 10 mL.Kg^−1^ of saline solution alone (control, C) or containing SDZ WAG 994 (SDZ, 60 µg.Kg^−1^), metyrapone (M, 1 mg.Kg^−1^), both SDZ WAG 994 and metyrapone (SDZ+M), or both SDZ WAG 994 and intralipid (3 mL.Kg^−1^) solution (SDZ+IL). The results are shown as mean+S.E.M. of 10 fish per treatment. Different letters indicate significant differences (*P*<0.05) among treatments.

Levels of metabolites assessed in plasma are shown in Fig. 2. FA (Fig. 2A) and triglyceride (Fig. 2B) levels decreased after SDZ treatment compared with all other groups. Total lipid levels (Fig. 2C) decreased after treatment with SDZ or SDZ +IL compared with the remaining groups, and the decrease was weaker in the SDZ +IL group. Glucose levels increased after treatment with SDZ +M compared with control and SDZ groups (Fig. 2D). Lactate levels increased after SDZ +IL treatment compared with SDZ +M (Fig. 2E). Cortisol levels were higher in the SDZ group than in all other groups (Fig. 2F).

**Figure 3:**
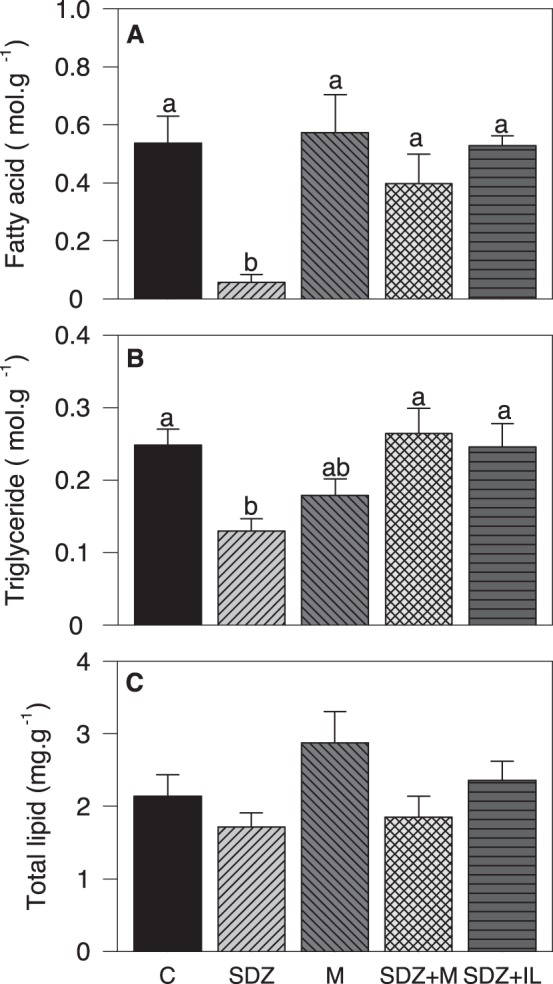
Changes in the levels of metabolites in hypothalamus after intraperitoneal treatment. Levels of fatty acid (A), triglyceride (B), and total lipid (C) in hypothalamus of rainbow trout after 6 h of intraperitoneal administration of 10 mL.Kg^−1^ of saline solution alone (control, C) or containing SDZ WAG 994 (SDZ, 60 µg.Kg^−1^), metyrapone (M, 1 mg.Kg^−1^), both SDZ WAG 994 and metyrapone (SDZ+M), or both SDZ WAG 994 and intralipid (3 mL.Kg^−1^) solution (SDZ+IL). The results are shown as mean+S.E.M. of 10 fish per treatment. Different letters indicate significant differences (*P*<0.05) among treatments.

In hypothalamus (Fig. 3) SDZ treatment decreased levels of FA (vs. all other groups) and triglycerides (vs. control, SDZ +M and SDZ +IL groups) whereas no significant changes were noted for total lipid levels.

**Figure 4:**
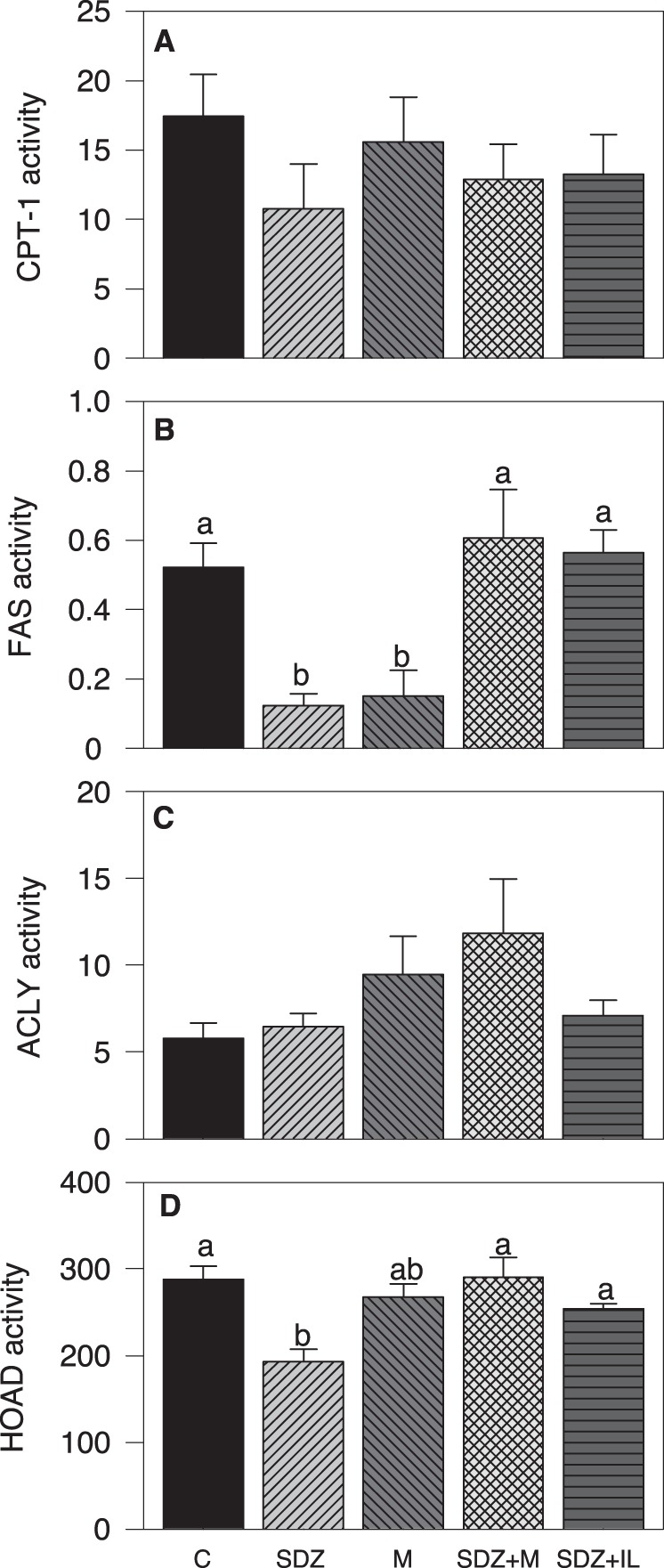
Changes in the activity of enzymes in hypothalamus after intraperitoneal treatment. Activities (mU.mg^−1^ protein) of CPT-1 (A), FAS (B), ACLY (C), and HOAD (D) in hypothalamus of rainbow trout after 6 h of intraperitoneal administration of 10 mL.Kg^−1^ of saline solution alone (control, C) or containing SDZ WAG 994 (SDZ, 60 µg.Kg^−1^), metyrapone (M, 1 mg.Kg^−1^), both SDZ WAG 994 and metyrapone (SDZ+M), or both SDZ WAG 994 and intralipid (3 mL.Kg^−1^) solution (SDZ+IL). The results are shown as mean+S.E.M. of 10 fish per treatment. Different letters indicate significant differences (*P*<0.05) among treatment.

Enzyme activities assessed in hypothalamus are shown in Fig. 4. FAS activity was lower in the SDZ and M groups compared with the remaining groups (Fig. 4B). HOAD activity was lower in the SDZ group than in control, SDZ +M, and SDZ +IL groups (Fig. 4D). We did not observe significant differences for CPT-1 (Fig. 4A) and ACLY (Fig. 4C) activities.

RNA abundance of transcripts related to fatty acid sensing in hypothalamus is shown in Table 2. FAT/CD36 level was lower in the SDZ group compared with control, M, and SDZ +IL groups. ACC level increased in the M group compared with control, SDZ +M, and SDZ +IL groups. ACLY level increased after M treatment compared with control, SDZ, and SDZ +IL treatments. CPT-1c level decreased in the SDZ group compared with control and SDZ +IL groups. Levels of CPT-1d in the SDZ group were lower than in control, M, and SDZ +IL groups. FAS level increased in the group treated with SDZ compared with all other groups. MCD values were lower in the SDZ group than in control and SDZ +IL groups. UCP2a values were higher in the SDZ group than in control, SDZ +M, and SDZ +IL groups whereas those of SDZ +IL group were lower than in all other groups. The level of SUR-like was lower in the M and SDZ +M groups than in all other groups. PPARα level increased after treatment with M or SDZ +M compared with all other groups. CART level decreased after treatment with SDZ or SDZ +M compared with control and SDZ +IL groups. POMC-A1 level decreased after treatment with SDZ or SDZ +M compared with control and SDZ +IL groups with the decrease being more important for SDZ +M; the value of SDZ +IL was higher than in all other groups. Finally, no significant changes were apparent for CS, Kir6.x-like, LXRα, and NPY.

**Table 2:** Relative mRNA abundance of transcripts related to fatty acid sensing in hypothalamus of rainbow trout after 6 h of intraperitoneal administration of 10 mL.Kg^−1^ of saline solution alone (control, C) or containing SDZ WAG 994 (SDZ, 60 µg.Kg^−1^), metyrapone (M, 1 mg.Kg^−1^), both SDZ WAG 994 and metyrapone (SDZ+M), or both SDZ WAG 994 and intralipid (3 mL.Kg^−1^) solution (SDZ+IL).

	C	SDZ	M	SDZ+M	SDZ+IL
*Fatty acid transport*					
FAT/CD36	1±0.05a	0.63±0.04b	1.13±0.09a	0.89±0.12ab	1.23±0.08a
*Fatty acid metabolism*					
ACC	1±0.16a	1.15±0.12ab	1.80±0.25b	0.99±0.08a	1.06±0.16a
ACLY	1±0.11a	1.07±0.11a	1.70±0.14b	1.30±0.20ab	0.99±0.14a
CPT-1c	1±0.08a	0.62±0.05b	0.90±0.09ab	0.69±0.10ab	1.15±0.11a
CPT-1d	1±0.06ab	0.57±0.04c	0.96±0.12ab	0.64±0.08bc	1.24±0.11a
CS	1±0.16	0.88±0.12	1.46±0.20	0.93±0.16	1.15±0.12
FAS	1±0.08a	1.58±0.09b	1.17±0.12a	1.16±0.09a	1.14±0.13a
MCD	1±0.08a	0.57±0.06b	0.75±0.13ab	0.72±0.05ab	1.43±0.23a
*Mitochondrial uncoupling*					
UCP2a	1±0.12a	1.51±0.10b	1.34±0.14a	0.82±0.14a	0.47±0.07c
*K_ATP_ channel*					
Kir6.x-like	1±0.13	0.98±0.08	0.92±0.11	0.86±0.09	1.40±0.21
SUR-like	1±0.12a	0.81±0.07a	0.58±0.07b	0.49±0.03b	1.03±0.10a
*Transcription factors*					
LXRα	1±0.13	0.73±0.06	1.22±0.16	0.86±0.09	1.03±0.12
PPARα	1±0.15a	1.02±0.13a	2.39±0.18b	1.88±0.14b	1.26±0.16a
SREBP1c	1±0.11	0.96±0.11	1.31±0.15	0.90±0.09	1.32±0.12
*Neuropeptides*					
CART	1±0.11a	0.53±0.13b	0.75±0.05ab	0.57±0.08b	1.13±0.11a
NPY	1±0.07	1.09±0.09	1.28±0.15	1.06±0.09	1.03±0.12
POMC-A1	1±0.06a	0.54±0.05b	0.75±0.08ab	0.37±0.06b	2.50±0.22c

Data represent mean of 5 measurements. Data is expressed with respect to the control group (expression results were normalized by EF-1α mRNA levels, which did not show changes among groups). Different letters indicate significant differences (*P*<0.05) among treatments.

RNA abundance of transcripts related to HPI axis in hypothalamus and head kidney is shown in Table 3. In hypothalamus, CRF value was higher in the SDZ group compared with all other groups whereas value of SDZ +IL group was higher than in M and SDZ +M groups. CRFBP levels increased after treatment with SDZ or M compared with control, SDZ +M, and SDZ +IL groups. 3βHSD level increased after treatment with SDZ compared with all other groups; values in the SDZ +M group were lower than in all other groups. 11βH level increased after treatment with SDZ or M compared with all other groups with the increase being higher for M group, and decreased after treatment with SDZ +M compared with all other groups. P450scc level increased in the group treated with SDZ compared with the remaining groups; the level in the M and SDZ +M groups was lower than in all other groups with the decrease being more important for SDZ +M. Finally, StAR value increased after treatment with SDZ compared with all other groups.

**Table 3:** Relative mRNA abundance of transcripts related to hypothalamus-pituitary-interrenal axis in hypothalamus and head kidney of rainbow trout after 6 h of intraperitoneal administration of 10 mL.Kg^−1^ of saline solution alone (control, C) or containing SDZ WAG 994 (SDZ, 60 µg.Kg^−1^), metyrapone (M, 1 mg.Kg^−1^), both SDZ WAG 994 and metyrapone (SDZ+M), or both SDZ WAG 994 and intralipid (3 mL.Kg^−1^) solution (SDZ+IL).

	C	SDZ	M	SDZ+M	SDZ+IL
*Hypothalamus*					
CRF	1±0.11a	1.74±0.10b	0.69±0.09a	0.63±0.07a	1.19±0.11a
CRFBP	1±0.09a	1.49±0.07b	1.88±0.17b	1.35±0.10a	1.01±0.12a
*Head kidney*					
3βHSD	1±0.17a	1.77±0.12b	1.30±0.15a	0.32±0.03c	0.86±0.14a
11βH	1±0.13a	1.91±0.14b	3.17±0.39c	0.31±0.09d	1.28±0.20a
P450scc	1±0.15a	1.68±0.07b	0.52±0.11c	0.08±0.01d	1.08±0.15a
StAR	1±0.18a	1.92±0.13	1.23±0.10a	0.77±0.09a	1.26±0.11a

Data represent mean of 5 measurements. Data is expressed with respect to the control group (expression results were normalized by EF-1α mRNA levels, which did not show changes among groups). Different letters indicate significant differences (*P*<0.05) among treatments.

## Discussion

### Lowering circulating FA levels in rainbow trout decreased anorexigenic potential in hypothalamus and increased food intake

Treatment with SDZ was effective in reducing not only circulating FA levels but also those of triglyceride and total lipid, which validates the experimental design. These changes are similar to those reported in rat after similar treatment [22], [29], [47]. There are no comparable references available in fish, though in zebrafish bezafibrate treatment was also able to decrease plasma triglyceride and cholesterol levels [48]. Moreover, the presence of intralipid was able to counteract the action of SDZ in rainbow trout resulting in levels of FA and triglyceride similar to those of controls, again in agreement with that reported in rat [22].

Food intake increased in fish treated with SDZ. This allows us to suggest, for the first time in fish, that hypothalamus sense the decreased levels of FA resulting in an orexigenic response stimulating food intake. We cannot exclude the possibility that SDZ could have a direct effect on food intake independent of that elicited by the inhibition of lipolysis. However, the finding that the presence of intralipid counteracted the increased food intake suggest that the effect of reduced FA levels is more likely. This increased food intake is in agreement with that reported in different fish species, including rainbow trout, after feeding diets with low lipid content [6], [8], [9], [10], [24], [25]. Furthermore, the food intake response is the opposite of that observed when the same species was treated with specific FA such as oleate or octanoate [14]. Changes elicited in the levels of circulating lipids relate to changes observed in hypothalamus since SDZ treatment decreased levels of FA and triglyceride, and levels recovered those of controls after additional treatment with intralipid. Therefore, changes in circulating metabolites in plasma are translated into changes in metabolite levels in hypothalamus thus supporting the FA sensing capacity of that tissue not only to increased levels of FA as previously described [14], [15], [16] but also to decreased levels of circulating FA as herein reported. Accordingly, we evaluated changes in parameters related to putative FA sensing systems in hypothalamus in order to relate them to changes observed in food intake.

SDZ treatment affected the FA sensing system related to FA metabolism since it decreased the activity of FAS and HOAD as well as mRNA abundance of CPT1c, CPT1d, and MCD and increased mRNA abundance of FAS. Furthermore, the presence of intralipid counteracted these changes. We have previously observed that this system responds to increased levels of FA with decreased lipogenic potential and decreased capacity of FA oxidation [14], [15], [16]. Therefore, we expected that changes observed in parameters related to FA metabolism would be different than those observed after increasing levels of specific FA such as oleate or octanoate [14]. Accordingly, we observed opposite changes for FAS activity and mRNA abundance as well as for mRNA abundance of CPT1c and MCD. However, other parameters that displayed changes in situations of increased circulating levels of oleate or octanoate [14], [15], [16] such as CPT-1 and ACLY activities and mRNA abundance of ACC, ACLY, and CS did not display any change in the present study. The responses observed suggest that the FA sensing system related to FA metabolism only respond partially to decreased circulating levels of FA. There are no similar studies available in fish hypothalamus [13] whereas in peripheral tissues like liver lipogenic potential is up-regulated when fish were fed with diets with low lipid content [49].

The putative FA sensing system related to FA transport through FAT/CD36 and subsequent modulation of transcription factors partially responded to decreased circulating levels of FA since several parameters did not show changes compared with controls (mRNA abundance of PPARα, SREBP1c, and LXRα). Only mRNA abundance of FAT/CD36 decreased after SDZ treatment. This change is the opposite of that previously observed when FA levels increased [14] suggesting a decreased potential for binding capacity of FAT/CD36 in parallel with the decreased circulating FA levels.

As for the putative FA sensing system related to K_ATP_ channel and mitochondrial activity, Kir6.x-like and SUR-like mRNA abundance displayed no changes when circulating FA levels decreased. However, levels of UCP2a mRNA were higher in the group treated with SDZ than in controls, a change opposite of that described when FA levels increased [14], [15].

Despite the relatively few changes observed in the FA sensing systems evaluated, mRNA abundance of anorexigenic peptides (POMC-A1 and CART) clearly decreased in hypothalamus of fish treated with SDZ. This response is the opposite of that observed under conditions of increased circulating FA levels where the abundance of these transcripts increased [13]. Moreover, the increase observed in the mRNA abundance of POMC-A1 in the SDZ+IL group could be associated with the returning of food intake in that group to values similar of those in control group. Furthermore, we did not observe significant changes in NPY mRNA abundance when FA levels decreased, in contrast with the decrease observed when FA levels increased [14], [15]. These changes suggest a decreased anorexigenic potential in hypothalamus of SDZ-treated fish, which is in agreement with changes noted in food intake. However, CRF mRNA levels increased under the same conditions what considering the anorexigenic nature of this peptide suggest a more complex relationship between changes in mRNA levels of neuropeptides and food intake.

Therefore, it seems that the decrease in circulating FA levels induced by SDZ treatment resulted in decreased levels of FA in hypothalamus but only few of the parameters involved in putative FA sensing systems addressed in hypothalamus changed accordingly. However, since the expression of several neuropeptides and, more important, food intake changed under those conditions, we can suggest, for the first time in non-mammalian vertebrates, that central capacity for sensing decreased FA levels exists in fish. However, this modulation of FA sensors is not exactly the opposite of that observed when FA levels increase [14], [15], [16]. The difference in the response of these systems to increased or decreased FA levels could be due to different factors. Any of these factors could be: i) the involvement of another putative FA sensing mechanism, such as that mediated by PKC-θ [2], or ii) the fact that we evaluated the decrease of FA in general whereas in the other studies carried out in rainbow trout [14], [15], [16] we evaluated the increase of specific FA, such as oleate and octanoate. In fact, in a recent study in rat, the counter-regulatory response to decreased FA levels depends on the type of FA [23].

### The activation of the HPI axis is probably involved in the FA counter-regulatory response to decreased levels of FA

In mammals, different hormones of lipolytic action such as growth hormone [50], epinephrine [51], glucagon [52] or glucocorticoids [21], [22], [23] were associated with the counter-regulatory response to decreased levels of FA. In the present study, we have evaluated the possibility that cortisol (main glucocorticoid in fish) could be involved in the counter-regulatory response observed in rainbow trout.

SDZ treatment induced an increase in circulating levels of cortisol, which was not observed in the SDZ+IL group. This allows us to suggest that the activation of the HPI axis is involved in the response to decreased levels of FA. This response is comparable to that observed in rat where the presence of intralipid also counteracted the decrease in circulating FA levels induced by SDZ treatment [22]. We have also monitored in head kidney the mRNA abundance of proteins involved in cortisol synthesis such as 11βH, 3βHSD, P450scc and StAR. Abundance of these transcripts increased after treatment with SDZ, and values returned to normality when fish were co-treated with SDZ and intralipid. In rainbow trout up-regulation of these transcripts corresponds with enhanced cortisol levels in plasma [53], [54]. Therefore, changes observed in these transcripts agree well with those of cortisol levels. Furthermore, we have also monitored hypothalamic CRF and CRFBP mRNA abundance that in the same species are normally changing in parallel with the levels of cortisol in plasma under short-term periods [55]. SDZ treatment increased levels of both transcripts and, once more, the presence of intralipid counteracted such elevation. Interestingly, an inverse relationship between plasma glucose and FA was observed in plasma in agreement with that seen in many fish species under stress situations in which the HPI axis is activated [56]. Altogether, these results suggest that decreased circulating levels of FA activate the HPI axis. However, we cannot discard a direct action of SDZ on cortisol synthesis.

Further support of the involvement of the HPI axis comes from results obtained in the groups treated with metyrapone. Metyrapone is an inhibitor of 11βH, and its treatment resulted in decreased circulating levels of cortisol in previously stressed fish [27], [28], [30] or no changes if fish were not previously stressed [57], [58], as observed in the present study. A glucocorticoid feedback response (i.e., increased CRF mRNA levels in response to decreased cortisol levels) has been demonstrated in rainbow trout when plasma cortisol levels changed after metyrapone treatment [27], [59]. Thus, the lack of changes in cortisol levels in the M group is also reflected by the absence of changes in hypothalamic CRF mRNA levels. Changes observed in hypothalamus of metyrapone-treated fish (up-regulation of ACC, ACLY and PPARα mRNA abundance) generally fit with those previously observed in catfish brain [58] where increased lipogenic capacity occurred after metyrapone treatment. However, since metyrapone treatment alone induced changes in several parameters assessed in hypothalamus and head kidney, we cannot discard that several of the effects could be attributable to an interactions between SDZ and metyrapone.

Based on the comparison between SDZ and SDZ+M groups, metyrapone treatment was effective in counteracting the increased cortisol levels elicited by SDZ treatment. This effect of metyrapone is in agreement with that previously observed in the same species under different stressful conditions [27], [28], [30]. Furthermore, the treatment with both metyrapone and SDZ induced in plasma changes in parameters in a way that values were comparable to those of the control group and different than those of the SDZ group, such as for plasma FA, triglyceride or total lipid levels. This return to normality is also evident in parameters assessed in hypothalamus where FA and triglyceride levels that decreased in the SDZ group turned back to normal values in the group treated with SDZ and metyrapone. In parameters related to the HPI axis, an effective blockade of its functioning was apparent as demonstrated by the strong decrease observed in transcript abundance of the four proteins related to cortisol metabolism assessed in head kidney in the SDZ+metyrapone group. These changes are associated with those observed in the levels of cortisol in plasma, which were similar to those of the control group and lower than in the SDZ-treated group. Further support comes from changes observed in CRF and CRFBP mRNA abundance in the hypothalamus where a significant inhibition of the response observed with the SDZ treatment was evident. Altogether, these results suggest us that the HPI axis is involved in the response induced by decreased circulating FA levels produced by SDZ treatment.

The remaining parameters assessed in hypothalamus related to FA sensing displayed in general in the group treated with SDZ and metyrapone values similar to those of controls and different than those elicited by SDZ treatment alone. Thus, metyrapone treatment effectively counteracted changes elicited by SDZ treatment in a way similar to those elicited by the presence of intralipid. Therefore, we suggest that the changes observed in FA sensing mechanisms involved in the counter-regulatory orexigenic response in hypothalamus could relate to the activation of the HPI axis. Since increased CRF production in hypothalamus activates this axis, we could speculate that CRF is modulating the activity of hypothalamic neurons that integrate metabolic information resulting in altered production of anorexigenic and orexigenic neuropeptides and finally in the food intake response, in a way comparable to that previously suggested for glucosensing in the same species [60]. However, blocking the HPI axis with metyrapone did not alter the decreased anorectic potential deduced by the down regulation observed in POMC-A1 and CART transcripts by SDZ treatment in a way similar to the lack of effect of metyrapone treatment on mRNA abundance of NPY in hypothalamus of stressed rainbow trout [59]. Moreover, stress situations and factors related to the HPI axis such as CRF have been demonstrated to be clearly anorexigenic in several fish species including rainbow trout [61], [62] and a clear increase in mRNA levels of CRF was also observed in hypothalamus of SDZ-treated fish. Therefore, the modulation of food intake control by HPI activation is complex and other factors besides those herein assessed, are likely involved.

## Conclusions

In summary, we have obtained evidence, for the first time in fish (and in a non-mammalian vertebrate), about the existence of a counter-regulatory response to a fall in circulating FA levels. The response is apparently associated with food intake control and the activation of HPI axis. Thus, the decrease in circulating levels of FA in rainbow trout induces an increase in food intake that is associated with the decrease of the anorexigenic potential in hypothalamus and with changes in several parameters related to putative FA-sensing mechanisms in hypothalamus. The treatment with intralipid counteracted these changes. The decrease in FA levels apparently induces a counter-regulatory response in rainbow trout in which the activation of the HPI axis is likely involved. This activation probably not related to the control of food intake through FA sensor systems [13] but to the modulation of lipolysis in peripheral tissues to restore FA levels in plasma. This counter-regulatory response initiated in the hypothalamus, probably through changes in CRF, and this activation would arrive to peripheral tissues such as liver where metabolic changes would occur accordingly. However, we cannot exclude the possibility that i) the fall in plasma FA was sensed outside the brain, and the information be transmitted via an afferent neural pathway or a humoral factor, or ii) that other lipolytic hormones in fish such as GH, glucagon or catecholamines [63], [64], [65], [66], [67] could be involved in the FA counter-regulatory response. All these possibilities clearly deserve further studies.
